# Cardiovascular risk estimation and statin adherence: a historical cohort study protocol

**DOI:** 10.3399/BJGPO.2024.0258

**Published:** 2025-06-04

**Authors:** Samuel Finnikin, Brian Willis, Rani Khatib, Tim Evans, Tom Marshall

**Affiliations:** 1 Department of Applied Health Sciences, College of Medical and Dental Sciences, University of Birmingham, Birmingham, UK; 2 Cardiology Department, Leeds Teaching Hospitals NHS Trust, Leeds, UK; 3 Leeds Institute of Cardiometabolic Medicine, University of Leeds, Leeds, UK; 4 Office for Health Improvement and Disparities, Department of Health and Social Care, London, UK

**Keywords:** medication adherence, hydroxymethylglutaryl-CoA reductase inhibitors, shared decision making, general practitioners, primary healthcare

## Abstract

**Background:**

Adherence to statins for the primary prevention of cardiovascular disease (CVD) is low. There is evidence that some facets of the initiation consultation, or the initiating clinician, are associated with adherence. CVD risk estimation is fundamental to statin initiation and shared decision making (SDM), because the benefits of statins are proportional to CVD risk. Absence of a recorded CVD score before statin initiation therefore indicates that SDM is unlikely.

**Aim:**

To investigate whether SDM, using the CVD risk score as a proxy measure, is associated with adherence to statins and CVD outcomes.

**Design & setting:**

A retrospective cohort study using a database of English primary care records.

**Method:**

The cohort will include statin-naïve patients aged 40–84 years initiated on statins for primary prevention between 2017 and 2020, categorised by the presence or absence of a CVD risk score at statin initiation. Statin adherence and persistence will be determined from subsequent statin prescriptions. Multivariable modelling, accounting for potential confounders, will determine the association between a recorded CVD risk score and subsequent statin adherence and with statin persistence. A secondary analysis will investigate the relationship to subsequent CVD outcomes and death.

**Conclusion:**

This research uses a record of CVD risk score as a proxy for SDM, to investigate the link between SDM and medication adherence. It will shed light on the relationship between how the initiation consultation is performed and subsequent adherence and persistence with treatment.

## How this fits in

Although millions of prescriptions for statins are issued in the UK every month, a large proportion of those tablets are not taken as prescribed, and many patients discontinue prematurely. Shared decision making (SDM) at the initiation of statins is thought to contribute to better adherence, and QRISK score is a necessary component of SDM in this context. This research will investigate if SDM, using QRISK score as a proxy, is associated with greater adherence to statins. If this link is found, then this provides a new focus for quality improvement that could increase adherence to, and therefore utility of, statins.

## Introduction

Statins are the most commonly used medication in the UK and are primarily used for the primary prevention of cardiovascular disease (CVD). In order to optimise the use of statins, in the absence of other indications (such as chronic kidney disease or familial hypercholesterolaemia), patients should be offered this medication when their estimated CVD risk exceeds a threshold value. Since 2014, the National Institute for Health and Care Excellence (NICE) has recommended that statins should be offered to patients when their 10-year CVD risk (as estimated using QRISK^1^) exceeds 10%.^2^ To derive the sought cardiovascular benefit of statins, they need to be taken as prescribed and as a long-term treatment. Adherence to statins is, however, known to be low, with roughly half of people being non-adherent after a year.^3,4^ Poor adherence to statins used for primary prevention is associated with an increased risk of cardiovascular events.^5^

SDM is an accepted and preferential approach to medical decision making that involves thorough discussion of the potential risks and benefits of treatment options with a patient.^6^ It has been argued that adherence to medications could be improved through better SDM at initiation,^7–10^ and there is evidence that some facets of the initiation consultation, or the clinician doing the initiation, are associated with adherence.^11,12^ However, establishing a link between SDM and adherence in real-world data is difficult, not least because measuring SDM in practice is difficult to do.^13^

In order to engage in an SDM process, patients must be informed of the potential risks and benefits of the options being discussed, such as taking a statin.^14^ For this particular decision, the benefits of the treatment are well established and possible to individualise using cardiovascular risk calculators such as QRISK, since the potential benefit is proportional to the estimated CVD risk.^1,15^ Since optimal SDM about statins involves individualised risk estimation, and QRISK is the tool recommended by NICE, absence of a QRISK score on a patient’s record indicates SDM is unlikely to have taken place; conversely, presence of a QRISK score can only be treated as an indication that SDM may have taken place. Previous studies using The Health Improvement Network (THIN) database have shown that many patients are initiated on statins without there being a QRISK score recorded in the patient’s record. Moreover, when QRISK is not recorded, the principal predictor of the decision to start statins is the total cholesterol level, whereas, when it is recorded, the CVD risk estimate is the principal predictor.^16,17^ It is worth noting that some comorbidities (chronic kidney disease [CKD], type 1 diabetes, and familial hypercholesterolaemia) put people at much higher risk of CVD. Risk calculators are not recommended for use, and therefore cannot inform SDM, in people with these conditions.

The presence or absence of a record of a QRISK score is, therefore, an indicator of different decision-making processes. If a QRISK score is not coded in the patient’s record when statins are initiated, the evidence suggests CVD risk estimates are not discussed at the time of statin initiation and shared decision making is unlikely to have taken place. Conversely, a QRISK score coded at the time of statin initiation indicates that the necessary information required for SDM was available to the clinician during the consultation. Thus, it is reasonable to hypothesise that there is an association between the coding of a CVD risk estimate (where indicated) and SDM when statins are initiated for the primary prevention of CVD.

Having established an association between a QRISK score coded at the time of statin initiation and SDM, we can use coding of QRISK score as an indicator of SDM to investigate the association between SDM and statin adherence, and with clinical outcomes in a novel and accessible way. We can establish whether SDM (as assessed by QRISK coding) is associated with improved statin adherence. Using the same cohort, we can also investigate whether there is an association between SDM (as assessed by QRISK coding) and CVD outcomes and death given that CVD risk would be reduced by improved adherence to statins (through an increased lifetime exposure to statins).

### Objective

The objective of this study is to investigate whether evidence of SDM (as determined by the recording of QRISK code where indicated) at the point of statin initiation is associated with adherence to, and persistence with, statins and CVD outcomes.

## Method

This will be a cohort study using Clinical Practice Research Datalink (CPRD) Aurum^18^ (version Dec. 2023[2023.12.001]). All statin-naïve patients aged 40–84 years (inclusive) initiated on statins for primary prevention between 2017 and 2020 will be included. To only include patients where a QRISK calculation is appropriate, patients will be excluded if they have existing CVD (or a CVD event within 60 days of statin initiation to account for any coding delay), type 1 diabetes mellitus, CKD stages 3–5, or familial hypercholesterolaemia (either coded or presumed because of total cholesterol being ≥9.0 mmol/L). Previous research has shown that around a third of patients initiated on statins (in 2015 and 2016) had a QRISK recorded.^17^ This splits the population into two clear cohorts, those with a coded QRISK score and those without, allowing comparisons to be made.

The index date will be the date of the first statin prescription. Patients will be eligible for inclusion from the earliest of the following dates: study start date, registration date plus 1 year, and age 40; until the earliest of the following dates: age 85, study end date, CVD diagnosis, other excluding diagnosis, or recording of a contraindication for the prescribing of statins. Patients will also be excluded if they left the database within 2 years of the index date as a minimum of 2-year follow-up is required.

Patients will be followed up until the earliest of the following dates: study end period, exit from the database, development of CVD, or death.

Patients initiated on a statin but who go on to develop a contraindication to continuing statin therapy (coded allergy/contraindication, commenced on interacting medication, or developed abnormal liver function or liver disease) will be identified. As the development of a contraindication to continuing statins will be independent of the initiation consultation, non-adherence due to contraindications is likely to be consistent between patients with or without QRISK scoring.

Baseline characteristics and variables that may be associated with adherence will be identified: age, sex, Index of Multiple Deprivation, smoking status, ethnic group, BMI, comorbidities (hypertension, type 2 diabetes mellitus, atrial fibrillation, inflammatory arthritis, and severe enduring mental illness), lipid profile (last recorded before index date), QRISK (latest recorded in the period 60 days prior to initiation up until statin initiation), prescriber role, and statin type and dose. It is envisaged that the majority of QRISK scores will be using the QRISK2 algorithm, but QRISK3^19^ codes will be extracted as well and treated in the same way.

The main outcome will be adherence to statins identified through all statin prescriptions (type and duration of prescription in days) following initiation. Persistence with statin therapy will be a secondary measure. Prescriptions for other oral lipid-lowering therapies (LLTs: ezetimibe, bempedoic acid, and fibrates) will also be extracted as it is possible that a patient who is intolerant to statins, but who would like to continue LLT, could be switched to non-statin alternatives. Primary analysis will focus on statin prescriptions alone, but a secondary analysis will include other LLTs. Other LLT prescriptions alone will not satisfy the inclusion criteria for the study as these are not a recommended first-line therapy for primary prevention. Any lipid profiles recorded following initiation will be identified, as will the development of CVD (myocardial infarction, ischaemic stroke, transient ischaemic attack, peripheral vascular disease, or angina).

### Analysis

The primary outcome of adherence will be measured using the Medication Possession Ratio (MPR) for each completed year of follow-up.^20,21^ The quantity of drug prescribed, rather than the ‘days’ prescribed, will be used for the prescription duration as it was felt that this would provide more accurate data. Statins (and other LLTs) are prescribed as a once-daily dosing regimen but the number of ‘days’ entered by the clinician can be inconsistent with this. A sensitivity analysis will be undertaken only including patients where number of days is equal to the quantity of drug prescribed to check this assumption. Patients will be excluded from adherence analysis if they are only issued one prescription of a statin (but these will be included in persistence analysis). Patients will be classified as adherent if their MPR is greater than 0.8 in a 12-month period as this threshold is consistently used for research purposes.^22^ Adherence (MPR) will be calculated for each completed 12-month period of follow-up to assess for trends.

For the secondary outcome of persistence, patients will be identified as having discontinued statin therapy if there is no new prescription for 180 days after the expected end of their supply. This time period was chosen to allow for variation in prescribing practices and is consistent with existing research. Persistence will be calculated for patients who discontinue treatment prior to exiting the cohort and analysed. This will be defined as the time between initiation and the final prescription plus the duration of the last prescription.^22^ A secondary analysis will be performed including all oral LLTs prescribed following statin initiation.

Some patients may restart their medication after a period of discontinuation >180 days, but any prescriptions after a period of discontinuation will not be included in adherence and persistence analysis. This is based on the assumption that further discussion about the medication may have influenced the decision to recommence, so the link with the initiation consultation is less robust. It is noted that this may weaken the association with adherence and persistence as well as CVD outcomes as patients may be more adherent on restarting. However, as this is a secondary outcome of this study, this limitation is accepted.

Multivariable logistic regression modelling will be undertaken to establish the impact of variables on adherence in the first 12 months (as measured by MPR). Persistence with presence or absence of QRISK score will be the main variable of interest and prescriber ID a random effect. Mediator analysis will be undertaken to establish if between-prescriber variation in adherence is mediated by QRISK coding. Modelling will also be undertaken for each group separately (those with QRISK coding and those without) to establish if predictors of adherence are different between the groups. Statistical significance will be adjusted for multiple testing using the Bonferroni technique.^23^

Two time-to-event analyses using Cox regression for the outcomes of statin discontinuation and CVD outcomes and death will be performed. In both cases, QRISK coding will be the main predictor variable.

Prescribing data are electronic and recorded automatically, and therefore it is assumed that the absence of a prescription means that none was issued. If QRISK score is missing, where indicated, then it will be presumed to be absent. Previous research has shown that when QRISK2 is not recorded it does not seem to influence prescribing decisions, which provides validity to this assumption.^17^ Categories for missing data will be used for categorical variables. All analysis will be performed using StataSE (version 18).

## Discussion

We anticipate that the majority of the variables of interest will be well recorded within the database, allowing us to undertake a reliable analysis. It is worth noting that some follow-up period will fall into time affected by the COVID-19 pandemic, which may influence patient behaviour such as adherence and persistence. It is acknowledged that we can only infer a causal link between QRISK coding and adherence. However, because of the nature of adherence, any more granular causation will be difficult to establish, especially quantitatively, and this research will make a significant contribution to the evidence base. Similarly, the argument that links SDM with QRISK with adherence contains assumptions. However, the existing research underpins these assumptions, and it is felt that these data will give us an interesting and important insight into this topic.

## Data

Primary data is not available through the authors due to data sharing restrictions but is available to researchers through CPRD (subject to approvals). Code lists used in data extraction and processing are available on request from the corresponding author.
